# Supplementary material for: A genetic model of ivabradine recapitulates results from randomized clinical trials
Source: PLoS One. 2020 Jul 21;15(7):e0236193. doi: 10.1371/journal.pone.0236193 (PMC7373274; doi:10.1371/journal.pone.0236193)
Supplement: S1 Appendix — (DOCX) [file pone.0236193.s001.docx]

**A genetic model of ivabradine recapitulates results from randomized clinical trials**

Marc-André Legault B.Sc., Johanna Sandoval M.Sc., Sylvie Provost M.Sc., Amina Barhdadi Ph.D., Louis-Philippe Lemieux Perreault Ph.D., Sonia Shah Ph.D., R.Thomas Lumbers Ph.D., Simon de Denus B. Pharm., Ph.D., Benoit Tyl M.D., Jean-Claude Tardif M.D., Marie-Pierre Dubé Ph.D.

# Supplementary Methods

## Additional information on UK Biobank variable selection

Phenotype data based on a touchscreen-based questionnaire followed by a verbal interview with a trained nurse was gathered. Hospitalization records are also available through linkage to the Health Episode Statistics (HES). For this project we used data from hospitalization episodes between the beginning of the HES linkage (April 1^st^ 1997) and the last available date for the current data release (March 1^st^ 2016). The date and cause of death were also available from death records made available through linkage to the National Health Services records for England, Scotland and Wales. We defined clinically relevant variables based on combinations of self-reported diseases, operation codes and hospitalization or death record ICD9/ICD10 codes. For the definition of most variables, self-reported diseases were included. However, for myocardial infarction we noticed that many self-reported events were unsupported by HES data even though they occurred within the time period of the HES linkage. We used the baseline resting heart rate measurement (variable #102), prioritizing the manual reading (variable #95) if available and taking the average value if many readings were available. We used age at recruitment defined in variable #21022 and sex in variable #31. Many of the self-reported myocardial infarction events co-occurred with ICD10 codes for related but distinct disorders such as I25.1 (atherosclerotic heart disease), I20.0 (unstable angina) or R07.4 (chest pain) without diagnostic codes for myocardial infarction, suggesting ischemic disease without a myocardial infarction event. For this reason, we ignored self-reported events for myocardial infarction as well as for angina, unstable angina and coronary artery disease as participants may incorrectly report them. For prospective analyses we used time from first baseline assessment centre visit (extracted from variable #53) in years. The censure date was defined as the date of death or date of end of follow up. The end of follow-up date was set to 2016-03-01 for England and Wales and to 2015-11-30 for Scotland as defined in the UK Biobank documentation (<https://biobank.ctsu.ox.ac.uk/~bbdatan/death_cancer_report_Sept16.pdf>). Individuals were assigned to countries based on the location of the UK Biobank assessment centre visited for the baseline visit.

## UK Biobank additional genetic quality controls

All UK Biobank participants were previously genotyped using two similar arrays, the UK BiLEVE Axiom Array and the UK Biobank Axiom Array and genome-wide imputation was conducted using the Haplotype Reference Consortium as the main reference panel. Additional genetic quality control was done using pyGenClean version 1.8.3 [1]. Variants or individuals with more than 2% missing genotypes (per sample and per variant, respectively) were filtered out. The self-reported sex and the genetic sex based on sexual chromosome was compared and individuals with discrepancies or with aneuploidies were removed from the analysis. We only considered individuals of European descent for this study as they represent the major population in the UK Biobank. We used the computed principal components from the UK Biobank and defined a region in principal components space using individuals identified as “white British ancestry” as a reference population [2]. To avoid including related individuals, we used the kinship estimates from the UK Biobank and randomly selected an individual for pairs with a kinship coefficient > 0.0884. Based on this criteria, included individuals are expected to have a 3^rd^ degree relationships or more [2]. The resulting post QC dataset included 413,083 individuals. There were 1,165 available imputed variants located at the *HCN4* gene region (chr15:73,612,200-73,661,605 ± 200kb padding) with a MAF above 1% and that were bi-allelic.

## External summary statistics

The CARDIoGRAMplusC4D consortium published a 1000 Genomes based meta-analysis of myocardial infarction and CAD of 60,810 CAD cases [3] and a more recent meta-analysis that adds the UK Biobank and the MIGen / CARDIOGRAM exome chip study [4]. The main data release was based on the UK Biobank “soft” CAD definition that includes self-reported chronic ischemic heart disease and angina patients as well as the cases for the “hard” CAD definition of previous myocardial infarction or revascularization.

Two recent GWAS of atrial fibrillation were used. The first dataset was published by Nielsen *et al.* [5] and was based on 60,620 atrial fibrillation patients of European ancestry from six studies. The second dataset was published by Roselli *et al .*[6] and included 65,446 atrial fibrillation patients predominantly of European ancestry. This study conducted a trans-ethnic association analysis that also included participants of Japanese (12.5%), African American (1.3%) and Brazilian and Hispanic (1.3%) populations.

For heart failure, we summary association statistics from the HERMES case-control consortium including 47,309 cases and 910,014 controls [7].

Finally, for stroke, we obtained summary associated statistics from the MEGASTROKE consortium who conducted GWAS for many stroke subtypes including ischemic stroke, large artery stroke, cardioembolic stroke and small vessel strokes. There were two available datasets, one based on European individuals including 40,585 stroke cases and a trans-ethnic GWAS dataset including 67,162 stroke cases [8].

To provide insight into the overlap between samples from the external summary statistics and the UK Biobank, we summarized the shared number of cases and controls in S7 Table.

## Mendelian randomization

Mendelian randomization is a technique to infer the causal effect of an exposure such as heart rate on an outcome such as atrial fibrillation. Because genetic variants are randomly assigned at birth and are generally not influenced by the environment, they represent an unconfounded way of modulating the exposure that may be suitable for causal inference. Unfortunately, MR is susceptible to other methodological issues some of which can be accounted for in more sophisticated models. A commonly used model that assumes the classical instrument variable conditions is the inverse variance weighted (IVW) approach [9]. In this approach, the causal estimates for multiple variants are averaged with weights corresponding to the precision of the causal estimates without accounting for the possibility of invalid genetic instruments. The MR-Egger test is a similar approach that extends the IVW by allowing an intercept term that corresponds to directional pleiotropy [10]. Directional pleiotropy is detectable if the mean direct effect of genetic variants on the outcome is different from zero. The MR-Egger approach relaxes the exclusion-restriction (or IV3) assumption that requires genetic variants to be conditionally independent of the outcome given the exposure and covariates. Instead, MR-Egger requires the instrument strength independent of direct effect (InSIDE) assumption which stipulates that the effect of genetic variants on the exposure should be independent from the direct effects. MR-Egger is useful method, but the InSIDE assumption is hard to verify and is likely to fail in many biologically plausible scenarios leading to possibly biased estimates [11]. Moreover, the IVW and MR-Egger are greatly influenced by outlier variants as they are based on a linear regression of individual variant effects. A more recent set of MR methods further relax these assumptions and rely on the hypothesis that the largest set of variants with homogeneous effects is likely to represent the set of valid instrument variables. The contamination mixture method uses a mixture model to assign variants to a distribution of valid causal effects and a distribution of noisy variants with a null expected causal effect and a large variance [12]. Similarly, the Mendelian Randomization Pleiotropy RESidual Sum and Outlier (MR-PRESSO) iteratively eliminates variants whose effects are outliers when compared to the others until a homogeneous signal remains corresponding to the estimated causal effect [13]. These methods are interesting because they are less dependent on hard to verify assumptions, they are robust even when some invalid instruments are included, and they allow variants to be individually tested for their heterogeneity and further investigated.

For MR with the heart rate GRS, we used the two-stage method which is akin to the previously described IVW method, but uses individual level data [14]. Heart rate expressed in units of 10 bpm reduction was predicted based on a fitted model including the GRS as a continuous variable and covariates (age, sex and PCs). The predicted heart rates were then used in the second stage to estimate the causal effect on CAD, heart failure and atrial fibrillation using logistic regression adjusted for the same covariates. The standard errors for the causal effect were estimated using the percentile method based on 5,000 bootstrap resamples which is an empirical way of estimating standard errors without assuming their distribution. This approach does not account for violations of the instrument variable assumptions, but the estimates rely on a strong genetic instrument whose effect is closer to pharmacological effects than what is observed using individual genetic variants making extrapolations less problematic.

Analyses were performed with the “MendelianRandomization” R package (<https://cran.r-project.org/web/packages/MendelianRandomization/>) and MR-PRESSO (<https://github.com/rondolab/MR-PRESSO>).

## Bi-directional MR

Bi-directional MR is a method used to infer the direction of the causality between two traits [15]. Genetic variants are ascertained for their association with the first trait and used as an instrument variable to estimate its effect on the second trait. The procedure is then repeated with instruments for the second trait (with the first trait as the outcome). The estimated causal effects can then be compared, and the direction of effect can be elucidated. Here, we were interested in the causality between atrial fibrillation and heart failure, CAD and myocardial infarction. We used 11 genome-wide significant independent variants from the HERMES consortium and estimated the causal effect of heart failure on atrial fibrillation using these variants. The previously described MR methods were used for the bi-directional analysis as well. To test the effect of atrial fibrillation on heart failure, we selected uncorrelated variants that reached genome-wide significance in the Nielsen *et al.* study [5]. The selection was done using *grstools* and the 1000 Genomes Phase III Europeans as a reference panel for linkage disequilibrium. Variants with a linkage disequilibrium r^2^ above 0.15 were clumped together, keeping the most significant variant. The selection was stopped when no genome-wide significant variant remained leaving 152 variants to be used as genetic instruments.

The variants for the myocardial infarction genetic instrument were selected as for the atrial fibrillation instrument but using the CARDIoGRAMplusC4D summary statistics. A total of 31 variants were selected all of which were available in the atrial fibrillation summary statistics. For the CAD instrument, we used the 71 variants comprising the genetic risk score described in Verweij *et al.* [16].

## Genetic risk score for heart rate

For the construction of the heart rate Genetic Risk Score (GRS), we used the 64 genome-wide significant heart rate associated SNPs from Eppinga *et al.* [17] To ensure that there was no strand confusion due to ambiguous alleles (i.e. A/T and G/C SNPs), we compared the observed allele frequencies to the expected distribution using the 1000 Genomes Phase III Europeans as a reference panel. If the observed allele frequency fell in a 95% Clopper-Pearson confidence interval around the reference panel frequency estimate, the strand was considered to be validated and the SNP was used as-is. When variants had a minor allele frequency (MAF) above 40%, an unambiguous SNP in LD was automatically selected to avoid strand confusion as the frequencies were close to 50%. A total of 8 of the 10 ambiguous variants were validated based on allele frequency. The other 2 variants had a MAF above 40% and were replaced by tag SNPs in LD. The variant rs13165531 was replaced by rs6887889 (r^2^=1) and rs3951016 was replaced by rs9401060 (r^2^=0.91). GRS weights were adjusted by multiplying the r^2^ value for both SNPs. The final set of variants and their corresponding weights are shown in Supplementary Table 5. The software used to compute the genetic risk scores is publicly available at <https://github.com/legaultmarc/grstools>.

# References for Supplementary Material

1. Lemieux Perreault LP, Provost S, Legault MA, Barhdadi A, Dube MP. pyGenClean: efficient tool for genetic data clean up before association testing. Bioinformatics. 2013;29(13):1704-5. doi: 10.1093/bioinformatics/btt261. PubMed PMID: 23652425; PubMed Central PMCID: PMC3694635.

2. Bycroft C, Freeman C, Petkova D, Band G, Elliott LT, Sharp K, et al. The UK Biobank resource with deep phenotyping and genomic data. Nature. 2018;562(7726):203-9. Epub 2018/10/12. doi: 10.1038/s41586-018-0579-z. PubMed PMID: 30305743.

3. Nikpay M, Goel A, Won HH, Hall LM, Willenborg C, Kanoni S, et al. A comprehensive 1,000 Genomes-based genome-wide association meta-analysis of coronary artery disease. Nat Genet. 2015;47(10):1121-30. Epub 2015/09/08. doi: 10.1038/ng.3396. PubMed PMID: 26343387; PubMed Central PMCID: PMCPMC4589895.

4. Nelson CP, Goel A, Butterworth AS, Kanoni S, Webb TR, Marouli E, et al. Association analyses based on false discovery rate implicate new loci for coronary artery disease. Nat Genet. 2017;49(9):1385-91. Epub 2017/07/18. doi: 10.1038/ng.3913. PubMed PMID: 28714975.

5. Nielsen JB, Thorolfsdottir RB, Fritsche LG, Zhou W, Skov MW, Graham SE, et al. Biobank-driven genomic discovery yields new insight into atrial fibrillation biology. Nat Genet. 2018;50(9):1234-9. Epub 2018/08/01. doi: 10.1038/s41588-018-0171-3. PubMed PMID: 30061737; PubMed Central PMCID: PMCPMC6530775.

6. Roselli C, Chaffin MD, Weng LC, Aeschbacher S, Ahlberg G, Albert CM, et al. Multi-ethnic genome-wide association study for atrial fibrillation. Nat Genet. 2018;50(9):1225-33. Epub 2018/06/13. doi: 10.1038/s41588-018-0133-9. PubMed PMID: 29892015; PubMed Central PMCID: PMCPMC6136836.

7. Shah S, Henry A, Roselli C, Lin H, Sveinbjornsson G, Fatemifar G, et al. Genome-wide association and Mendelian randomisation analysis provide insights into the pathogenesis of heart failure. Nat Commun. 2020;11(1):163. Epub 2020/01/11. doi: 10.1038/s41467-019-13690-5. PubMed PMID: 31919418; PubMed Central PMCID: PMCPMC6952380.

8. Malik R, Chauhan G, Traylor M, Sargurupremraj M, Okada Y, Mishra A, et al. Multiancestry genome-wide association study of 520,000 subjects identifies 32 loci associated with stroke and stroke subtypes. Nat Genet. 2018;50(4):524-37. Epub 2018/03/14. doi: 10.1038/s41588-018-0058-3. PubMed PMID: 29531354; PubMed Central PMCID: PMCPMC5968830.

9. Burgess S, Butterworth A, Thompson SG. Mendelian randomization analysis with multiple genetic variants using summarized data. Genet Epidemiol. 2013;37(7):658-65. Epub 2013/10/12. doi: 10.1002/gepi.21758. PubMed PMID: 24114802; PubMed Central PMCID: PMCPMC4377079.

10. Bowden J, Davey Smith G, Burgess S. Mendelian randomization with invalid instruments: effect estimation and bias detection through Egger regression. Int J Epidemiol. 2015;44(2):512-25. Epub 2015/06/08. doi: 10.1093/ije/dyv080. PubMed PMID: 26050253; PubMed Central PMCID: PMCPMC4469799.

11. Burgess S, Thompson SG. Interpreting findings from Mendelian randomization using the MR-Egger method. Eur J Epidemiol. 2017;32(5):377-89. Epub 2017/05/21. doi: 10.1007/s10654-017-0255-x. PubMed PMID: 28527048; PubMed Central PMCID: PMCPMC5506233.

12. Burgess S, Foley CN, Allara E, Staley JR, Howson JMM. A robust and efficient method for Mendelian randomization with hundreds of genetic variants. Nat Commun. 2020;11(1):376. Epub 2020/01/19. doi: 10.1038/s41467-019-14156-4. PubMed PMID: 31953392; PubMed Central PMCID: PMCPMC6969055.

13. Verbanck M, Chen CY, Neale B, Do R. Detection of widespread horizontal pleiotropy in causal relationships inferred from Mendelian randomization between complex traits and diseases. Nat Genet. 2018;50(5):693-8. Epub 2018/04/25. doi: 10.1038/s41588-018-0099-7. PubMed PMID: 29686387; PubMed Central PMCID: PMCPMC6083837.

14. Burgess S, Small DS, Thompson SG. A review of instrumental variable estimators for Mendelian randomization. Stat Methods Med Res. 2017;26(5):2333-55. Epub 2015/08/19. doi: 10.1177/0962280215597579. PubMed PMID: 26282889; PubMed Central PMCID: PMCPMC5642006.

15. Brower MA, Hai Y, Jones MR, Guo X, Chen YI, Rotter JI, et al. Bidirectional Mendelian randomization to explore the causal relationships between body mass index and polycystic ovary syndrome. Hum Reprod. 2019;34(1):127-36. Epub 2018/11/30. doi: 10.1093/humrep/dey343. PubMed PMID: 30496407; PubMed Central PMCID: PMCPMC6295958.

16. Verweij N, Eppinga RN, Hagemeijer Y, van der Harst P. Identification of 15 novel risk loci for coronary artery disease and genetic risk of recurrent events, atrial fibrillation and heart failure. Sci Rep. 2017;7(1):2761. Epub 2017/06/07. doi: 10.1038/s41598-017-03062-8. PubMed PMID: 28584231; PubMed Central PMCID: PMCPMC5459820.

17. Eppinga RN, Hagemeijer Y, Burgess S, Hinds DA, Stefansson K, Gudbjartsson DF, et al. Identification of genomic loci associated with resting heart rate and shared genetic predictors with all-cause mortality. Nat Genet. 2016;48(12):1557-63. Epub 2016/11/01. doi: 10.1038/ng.3708. PubMed PMID: 27798624.

18. Swedberg K, Komajda M, Bohm M, Borer JS, Ford I, Dubost-Brama A, et al. Ivabradine and outcomes in chronic heart failure (SHIFT): a randomised placebo-controlled study. Lancet. 2010;376(9744):875-85. Epub 2010/08/31. doi: 10.1016/S0140-6736(10)61198-1. PubMed PMID: 20801500.

19. Fox K, Ford I, Steg PG, Tendera M, Ferrari R, Investigators B. Ivabradine for patients with stable coronary artery disease and left-ventricular systolic dysfunction (BEAUTIFUL): a randomised, double-blind, placebo-controlled trial. Lancet. 2008;372(9641):807-16. Epub 2008/09/02. doi: 10.1016/S0140-6736(08)61170-8. PubMed PMID: 18757088.

20. Fox K, Ford I, Steg PG, Tardif JC, Tendera M, Ferrari R, et al. Ivabradine in stable coronary artery disease without clinical heart failure. N Engl J Med. 2014;371(12):1091-9. Epub 2014/09/02. doi: 10.1056/NEJMoa1406430. PubMed PMID: 25176136.

21. Buniello A, MacArthur JAL, Cerezo M, Harris LW, Hayhurst J, Malangone C, et al. The NHGRI-EBI GWAS Catalog of published genome-wide association studies, targeted arrays and summary statistics 2019. Nucleic Acids Res. 2019;47(D1):D1005-D12. Epub 2018/11/18. doi: 10.1093/nar/gky1120. PubMed PMID: 30445434; PubMed Central PMCID: PMCPMC6323933.

22. Ramirez J, Duijvenboden SV, Ntalla I, Mifsud B, Warren HR, Tzanis E, et al. Thirty loci identified for heart rate response to exercise and recovery implicate autonomic nervous system. Nat Commun. 2018;9(1):1947. Epub 2018/05/18. doi: 10.1038/s41467-018-04148-1. PubMed PMID: 29769521; PubMed Central PMCID: PMCPMC5955978.

23. Ng E, Lind PM, Lindgren C, Ingelsson E, Mahajan A, Morris A, et al. Genome-wide association study of toxic metals and trace elements reveals novel associations. Hum Mol Genet. 2015;24(16):4739-45. Epub 2015/05/31. doi: 10.1093/hmg/ddv190. PubMed PMID: 26025379; PubMed Central PMCID: PMCPMC4512629.

24. Morris JA, Kemp JP, Youlten SE, Laurent L, Logan JG, Chai RC, et al. An atlas of genetic influences on osteoporosis in humans and mice. Nat Genet. 2019;51(2):258-66. Epub 2019/01/02. doi: 10.1038/s41588-018-0302-x. PubMed PMID: 30598549; PubMed Central PMCID: PMCPMC6358485.

25. Lutz SM, Cho MH, Young K, Hersh CP, Castaldi PJ, McDonald ML, et al. A genome-wide association study identifies risk loci for spirometric measures among smokers of European and African ancestry. BMC Genet. 2015;16:138. Epub 2015/12/05. doi: 10.1186/s12863-015-0299-4. PubMed PMID: 26634245; PubMed Central PMCID: PMCPMC4668640.

26. Ueta M, Sawai H, Shingaki R, Kawai Y, Sotozono C, Kojima K, et al. Genome-wide association study using the ethnicity-specific Japonica array: identification of new susceptibility loci for cold medicine-related Stevens-Johnson syndrome with severe ocular complications. J Hum Genet. 2017;62(4):485-9. Epub 2017/01/20. doi: 10.1038/jhg.2016.160. PubMed PMID: 28100913.

27. Ellinor PT, Lunetta KL, Albert CM, Glazer NL, Ritchie MD, Smith AV, et al. Meta-analysis identifies six new susceptibility loci for atrial fibrillation. Nat Genet. 2012;44(6):670-5. Epub 2012/05/01. doi: 10.1038/ng.2261. PubMed PMID: 22544366; PubMed Central PMCID: PMCPMC3366038.

28. Christophersen IE, Rienstra M, Roselli C, Yin X, Geelhoed B, Barnard J, et al. Large-scale analyses of common and rare variants identify 12 new loci associated with atrial fibrillation. Nat Genet. 2017;49(6):946-52. Epub 2017/04/19. doi: 10.1038/ng.3843. PubMed PMID: 28416818; PubMed Central PMCID: PMCPMC5585859.

29. Nolte IM, Munoz ML, Tragante V, Amare AT, Jansen R, Vaez A, et al. Genetic loci associated with heart rate variability and their effects on cardiac disease risk. Nat Commun. 2017;8:15805. Epub 2017/06/15. doi: 10.1038/ncomms15805. PubMed PMID: 28613276; PubMed Central PMCID: PMCPMC5474732.

30. den Hoed M, Eijgelsheim M, Esko T, Brundel BJ, Peal DS, Evans DM, et al. Identification of heart rate-associated loci and their effects on cardiac conduction and rhythm disorders. Nat Genet. 2013;45(6):621-31. Epub 2013/04/16. doi: 10.1038/ng.2610. PubMed PMID: 23583979; PubMed Central PMCID: PMCPMC3696959.

31. Winkler TW, Justice AE, Graff M, Barata L, Feitosa MF, Chu S, et al. The Influence of Age and Sex on Genetic Associations with Adult Body Size and Shape: A Large-Scale Genome-Wide Interaction Study. PLoS genetics. 2015;11(10):e1005378. Epub 2015/10/02. doi: 10.1371/journal.pgen.1005378. PubMed PMID: 26426971; PubMed Central PMCID: PMCPMC4591371.

32. Machiela MJ, Chanock SJ. LDlink: a web-based application for exploring population-specific haplotype structure and linking correlated alleles of possible functional variants. Bioinformatics. 2015;31(21):3555-7. Epub 2015/07/04. doi: 10.1093/bioinformatics/btv402. PubMed PMID: 26139635; PubMed Central PMCID: PMCPMC4626747.

33. Consortium CAD, Deloukas P, Kanoni S, Willenborg C, Farrall M, Assimes TL, et al. Large-scale association analysis identifies new risk loci for coronary artery disease. Nat Genet. 2013;45(1):25-33. Epub 2012/12/04. doi: 10.1038/ng.2480. PubMed PMID: 23202125; PubMed Central PMCID: PMCPMC3679547.

34. Myocardial Infarction G, Investigators CAEC, Stitziel NO, Stirrups KE, Masca NG, Erdmann J, et al. Coding Variation in ANGPTL4, LPL, and SVEP1 and the Risk of Coronary Disease. N Engl J Med. 2016;374(12):1134-44. Epub 2016/03/05. doi: 10.1056/NEJMoa1507652. PubMed PMID: 26934567; PubMed Central PMCID: PMCPMC4850838.
