# Supplementary material for: A genetic model of ivabradine recapitulates results from randomized clinical trials
Source: PLoS One. 2020 Jul 21;15(7):e0236193. doi: 10.1371/journal.pone.0236193 (PMC7373274; doi:10.1371/journal.pone.0236193)
Supplement: S1 Fig — (DOCX) [file pone.0236193.s002.docx]

| 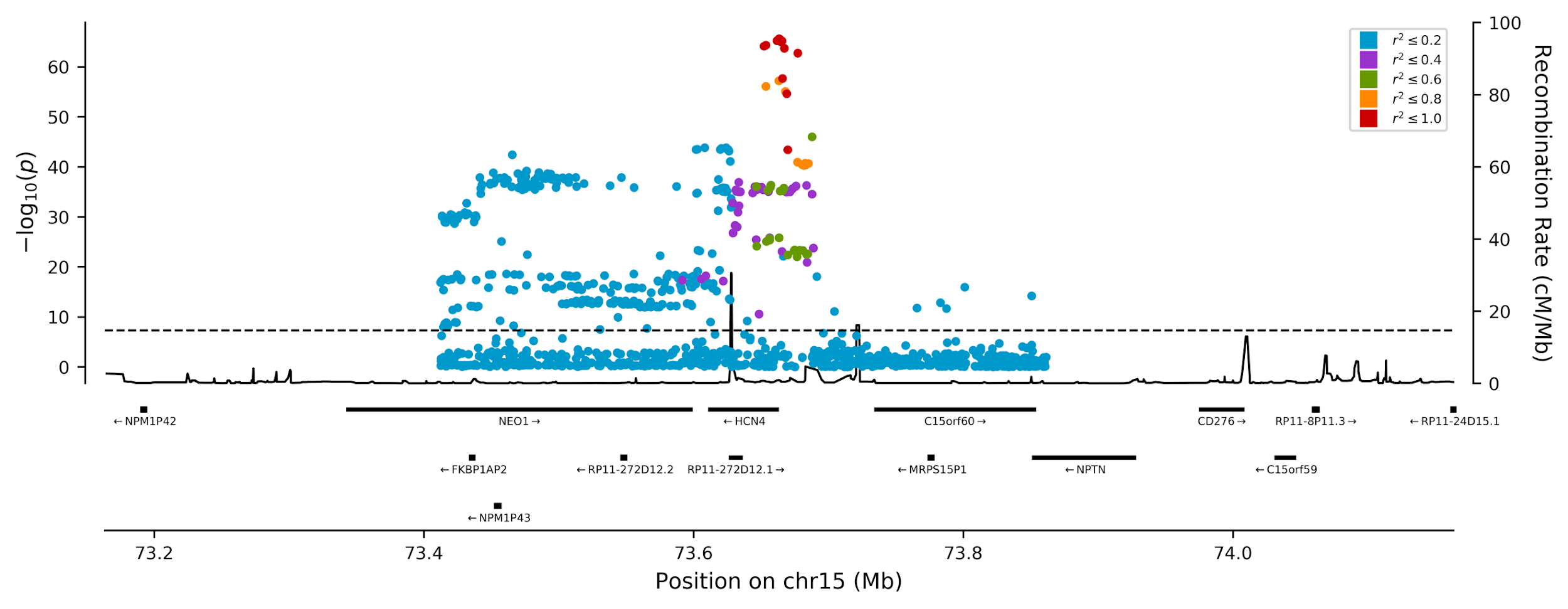 |
| --- |
| 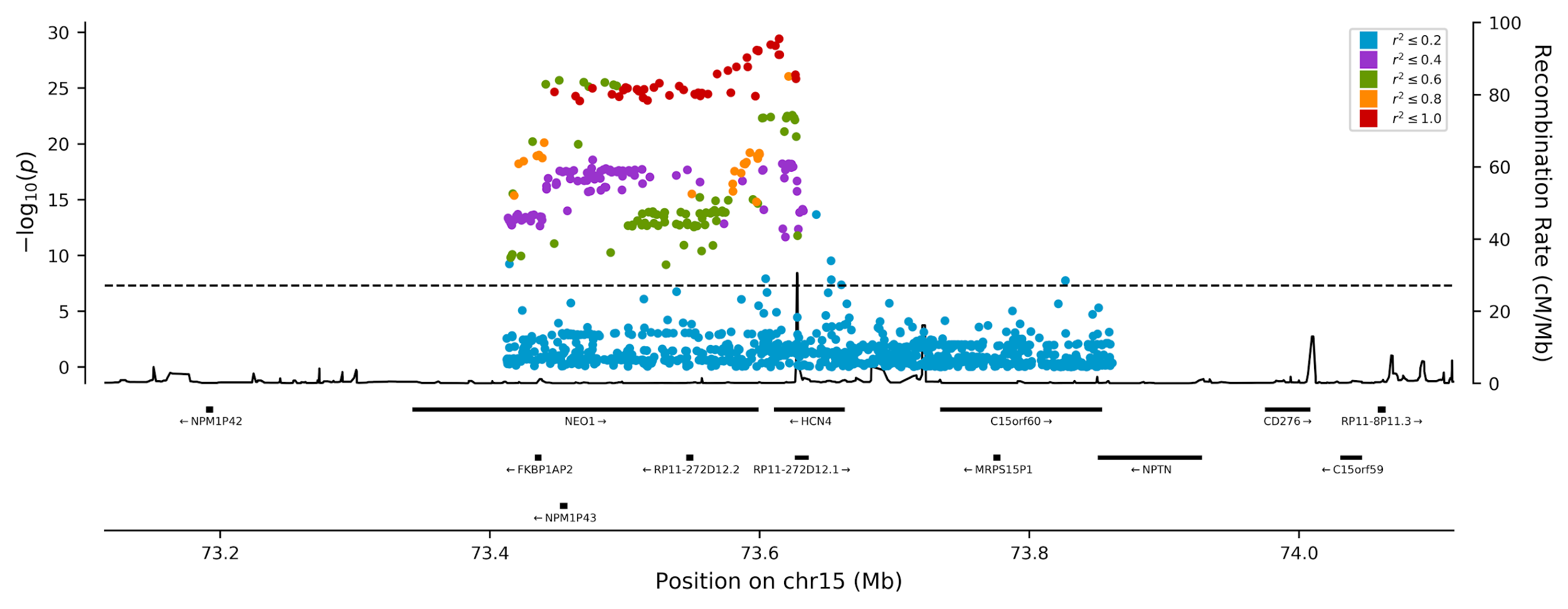 |
| 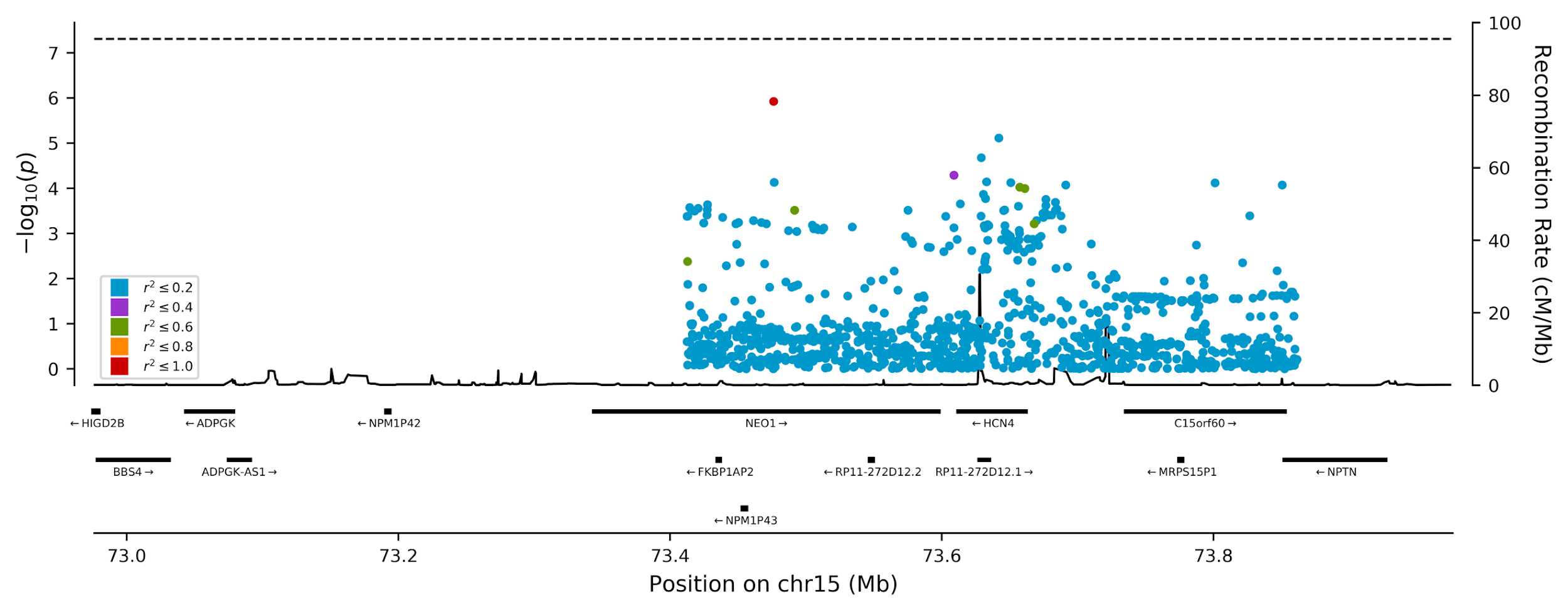 |

**S1 Figure.** Results from the stepwise forward regression using 1,165 variants in the *HCN4* region tested for association with heart rate in the UK Biobank, and adjusted for age, sex and the first 10 principal components. The lead variant identified was rs8038766, β = -0.574 (95% CI -0.640, -0.509), P-value=2.76×10^-66^ (results shown in the top panel). For the second stage, we conditioned on the lead variant from Stage 1 (rs8038766) and repeated the analysis. The lead variant identified in Stage 2 was rs3743496, β =-0.297 (95% CI -0.349, -0.246), P-value =3.96×10^-30^ (results shown in the second panel). We repeated the analysis again conditioning on the lead variants from both previous stages (rs8038766 and rs3743496), but no additional variant crossed the genome-wide significance threshold (third panel). The first y-axis shows the negative log10 of P-values, the second y-axis shows the recombination rate from HapMap reference samples (black line). Genes are displayed below the x-axis from Ensembl (build37), the degree of linkage disequilibrium (r^2^) of each genetic variant with the lead variant.
