# Supplementary material for: A genetic model of ivabradine recapitulates results from randomized clinical trials
Source: PLoS One. 2020 Jul 21;15(7):e0236193. doi: 10.1371/journal.pone.0236193 (PMC7373274; doi:10.1371/journal.pone.0236193)
Supplement: S2 Fig — (DOCX) [file pone.0236193.s003.docx]

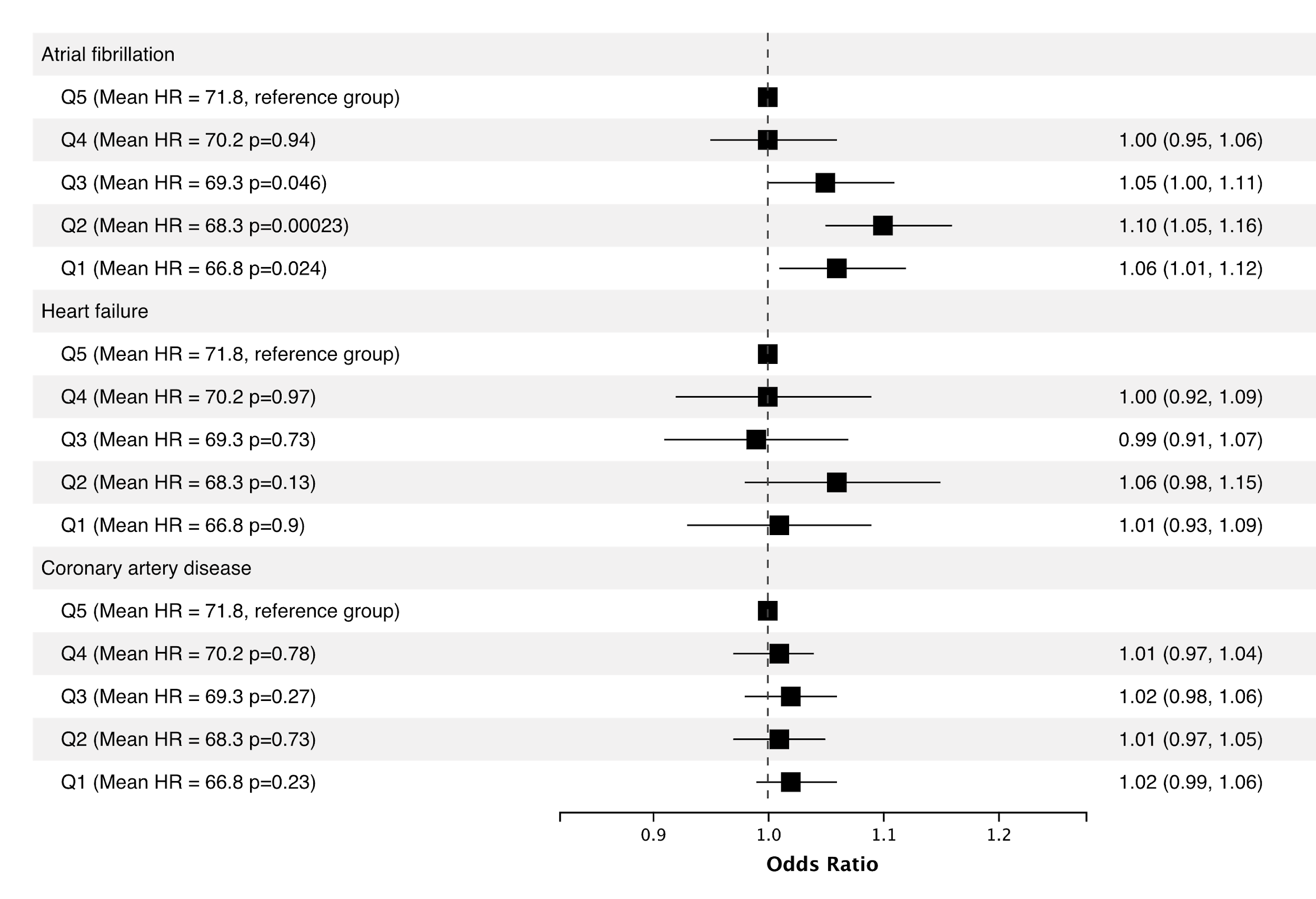


**S2 Figure**. Effect of heart rate genetic risk score groups based on quintiles on atrial fibrillation, heart failure and coronary artery disease in the UK biobank dataset. For every outcome, the highest heart rate group (determined by the 5^th^ quintile) is used as the reference group and the reported odds ratios are adjusted for age, sex and the first 10 principal components.
