# Supplementary material for: A genetic model of ivabradine recapitulates results from randomized clinical trials
Source: PLoS One. 2020 Jul 21;15(7):e0236193. doi: 10.1371/journal.pone.0236193 (PMC7373274; doi:10.1371/journal.pone.0236193)
Supplement: S1 Table — (DOCX) [file pone.0236193.s004.docx]

**S1 Table.** Summary of ivabradine cardiovascular outcomes trials.

| **Study / Intervention** | **Patient population** | **Main cardiovascular exclusions** | **Primary efficacy endpoint** | **Results** |
| --- | --- | --- | --- | --- |
| SHIFT [18]  2.5-7.5 mg bid versus placebo | - Resting heart rate >=70 bpm - Symptomatic chronic HF for at least 4 weeks - LVEF <= 35% - Recent hospitalization for worsening HF | - HF caused by congenital heart disease or primary severe valvular disease - Recent MI, atrial fibrillation or flutter | CV death or hospitalization for worsening HF | HR 0.82 (0.75, 0.90) p<0.0001. Effect driven by hospitalization for worsening HF.  Adverse events:  Atrial fibrillation 9% in ivabradine vs 8% in placebo (p=0.012). |
| BEAUTIFUL [19]  5-7.5 mg bid versus placebo | - Stable CAD (previous MI, PCI/CABG or angiographic evidence of obstruction of at least 50%) - LVEF <= 40% - Resting heart rate >= 65 bpm | - Recent revascularization or MI - Recent stroke or TIA - NYHA class IV HF - Implanted pacemaker, cardioverter or defibrillator - Valvular disease, SSS, sinoatrial block, severe hypertension | CV death or MI or hospitalization for worsening HF | Primary endpoint was not significant (p=0.94).  In a prespecified subgroup with baseline heart rate >= 70bpm, there was a reduction for ischemic endpoints, which led to conduct SIGNIFY. |
| SIGNIFY [20]  5-10 mg bid (treat to target of 55-60 bpm) versus placebo | - Stable CAD - No heart failure - Resting heart rate >= 70 bpm | - Patients with LVEF <= 40% | CV death or MI | Primary endpoint and its individual components were not significant  In patients with severe angina (>= CCS II) there was an increase of the primary endpoint with ivabradine HR 1.18 (1.03, 1.35) p=0.02. Individuals in this group also found better anginal symptom improvement on ivabradine  Atrial fibrillation was 5.3% in ivabradine vs 3.8% in placebo |

CABG, coronary artery bypass graft; CAD, coronary artery disease; CV, cardiovascular; HF, heart failure; HR, hazard ratio; LVEF, left ventricular ejection fraction; MI, myocardial infarction; PCI, percutaneous coronary intervention; TIA, transient ischemic attack
