# Supplementary material for: A genetic model of ivabradine recapitulates results from randomized clinical trials
Source: PLoS One. 2020 Jul 21;15(7):e0236193. doi: 10.1371/journal.pone.0236193 (PMC7373274; doi:10.1371/journal.pone.0236193)
Supplement: S2 Table — (DOCX) [file pone.0236193.s005.docx]

**S2 Table**. Self-reported, hospitalization (ICD10) and operation (OPCS) codes used to define clinical variables based on the UK Biobank available data.

|  | **Included codes** | | |
| --- | --- | --- | --- |
| **Variable** | Self-reported disease (variable #20002) | ICD9/10 for HES primary or secondary hospitalization codes or primary cause of death | Operations (OPCS) |
| **Angina** | - | ICD9: 413  ICD10: I20 |  |
| **Unstable angina** | - | ICD10: I20.0 (as the primary hospitalization code only to ensure acute event) |  |
| **Myocardial infarction** | - | ICD9: 410, 412, 411.0, 429.79  ICD10: I21, I22, I23, I25.2 |  |
| **Coronary artery disease** | - | ICD9: 410-414 (except 414.1)  ICD10: I20-I25 | K40, K41, K42, K43, K44, K45, K46, K49, K50, K75 |
| **Stroke (any)** | 1583, 1081, 1086, 1491 | ICD9: 430, 431, 434, 436  ICD10: I60, I61, I63, I64 |  |
| **Stroke - Ischemic** | 1583 | ICD9: 434, 436  ICD10: I63, I64 |  |
| **Atrial fibrillation** | 1471 | ICD9: 427.3  ICD10: I48 |  |
| **Heart failure** | 1076 | ICD9: 428, 425  ICD10: I50, I42 |  |
