# Supplementary material for: A genetic model of ivabradine recapitulates results from randomized clinical trials
Source: PLoS One. 2020 Jul 21;15(7):e0236193. doi: 10.1371/journal.pone.0236193 (PMC7373274; doi:10.1371/journal.pone.0236193)
Supplement: S3 Table — (DOCX) [file pone.0236193.s006.docx]

**S3 Table**. Results from the NHGRI-EBI GWAS catalog mapped to the *HCN4* gene [21]. LD with the lead independent heart-rate associated variants in the UK Biobank identified through forward stepwise conditional analysis are reported.

| **Variant and risk allele** | **Beta** | **P value** | **Trait** | **Study reference** | **LD with rs8038766 ^a^** | **LD with rs3743496 ^a^** |
| --- | --- | --- | --- | --- | --- | --- |
| rs7173389-T | 0.539 | 1.00E-32 | Resting heart rate | [17] | 1 | 0.022 |
| rs7173389-A | 0.528 | 2.00E-09 | Resting heart rate | [22] | 1 | 0.022 |
| rs8040516-T | 0.219 | 3.00E-06 | Nickel levels | [23] | 0.001 | 0.532 |
| rs74022964-T | 0.113 | 4.00E-36 | Atrial fibrillation | [5] | 0.956 | 0.022 |
| rs478438-G | 0.019 | 2.00E-09 | Heel bone mineral density | [24] | 0.237 | 0.050 |
| rs142859932-G | 0.487 | 3.00E-06 | Post bronchodilator FEV1 | [25] | - | - |
| rs16957893-C | 1.72 | 2.00E-08 | Cold medicine-related Stevens-Johnson syndrome/toxic epidermal necrolysis (SJS/TEN) with severe ocular complications | [26] | 0.025 | 0.004 |
| rs7164883-G | 0.17 | 3.00E-17 | Atrial fibrillation | [27] | 0.978 | 0.022 |
| rs7183206-A | 0.12 | 8.00E-12 | Atrial fibrillation | [28] | 0.956 | 0.018 |
| rs2680344-A | 0.024 | 5.00E-11 | Heart rate variability traits (SDNN) | [29] | 0.575 | 0 |
| rs2680344-A | 0.024 | 3.00E-11 | Heart rate variability traits (SDNN) | [29] | 0.575 | 0 |
| rs2680344-A | 0.032 | 1.00E-10 | Heart rate variability traits (RMSSD) | [29] | 0.575 | 0 |
| rs2680344-A | 0.046 | 3.00E-06 | Heart rate variability traits (pvRSA/HF) | [29] | 0.575 | 0 |
| rs4489968-T | 0.513 | 4.00E-20 | Heart rate | [30] | 1 | 0.022 |
| rs7172038-G | 0.10 | 2.00E-27 | Atrial fibrillation | [6] | 0.993 | 0.022 |
| rs74022964-T | 0.10 | 1.00E-27 | Atrial fibrillation | [6] | 0.956 | 0.022 |
| rs11072405-A | 0.021 | 1.00E-08 | Waist-to-hip ratio adjusted for BMI | [31] | 0.007 | 0.621 |
| rs11072405-A |  | 5.00E-07 | Waist-to-hip ratio adjusted for BMI×sex×age interaction (4df test) | [31] | 0.007 | 0.621 |

**^a^** LD measurements (r^2^) are for individuals of European (EUR) descent from the 1000 Genomes Project (phase 3) and were obtained using LDlink [32].
