# Supplementary material for: A genetic model of ivabradine recapitulates results from randomized clinical trials
Source: PLoS One. 2020 Jul 21;15(7):e0236193. doi: 10.1371/journal.pone.0236193 (PMC7373274; doi:10.1371/journal.pone.0236193)
Supplement: S4 Table — (DOCX) [file pone.0236193.s007.docx]

**S4 Table**. Variants and weights used for the computation of the heart rate GRS.

| **Variant** | **Chr.** | **Position** | **Reference allele** | **Risk allele** | **P value** | **Effect** |
| --- | --- | --- | --- | --- | --- | --- |
| rs145358377 | 1 | 6272136 | G | GA | 1.94E-11 | -0.259 |
| rs272564 | 1 | 45012273 | A | C | 4.51E-21 | 0.351 |
| rs2152735 | 1 | 87893132 | G | A | 7.23E-18 | -0.306 |
| rs41317993 | 1 | 207961732 | G | A | 5.42E-31 | 0.630 |
| rs11454451 | 1 | 217722890 | C | CT | 1.29E-11 | 0.256 |
| rs1260326 | 2 | 27730940 | T | C | 4.29E-16 | -0.275 |
| rs12713404 | 2 | 60006705 | G | T | 9.33E-09 | -0.199 |
| rs564190295 | 2 | 175547672 | G | GCCGCCGCCCCC | 4.95E-10 | -0.355 |
| rs151041685 | 2 | 179725237 | G | T | 7.86E-75 | 1.061 |
| rs62172372 | 2 | 188242369 | A | G | 5.99E-16 | 0.337 |
| rs907683 | 2 | 220299541 | G | T | 1.02E-20 | -0.334 |
| rs4608502 | 2 | 228134155 | T | C | 1.85E-12 | 0.249 |
| rs13002735 | 2 | 232268884 | A | C | 1.29E-17 | -0.331 |
| rs41312411 | 3 | 38621237 | C | G | 1.34E-11 | -0.320 |
| rs3749237 | 3 | 49770032 | G | A | 3.09E-13 | 0.258 |
| rs2358740 | 3 | 53455569 | G | T | 3.58E-09 | -0.208 |
| rs1483890 | 3 | 69410725 | A | G | 2.54E-15 | 0.284 |
| rs11920570 | 3 | 122090102 | G | A | 5.18E-13 | 0.268 |
| rs7612445 | 3 | 179172979 | G | T | 2.41E-24 | -0.428 |
| rs12501032 | 4 | 23951018 | C | G | 1.83E-15 | 0.288 |
| rs6845865 | 4 | 148974602 | T | C | 2.25E-14 | -0.342 |
| rs6887889 (tag for rs13165531) | 5 | 30893205 | T | G | 3.57E-09 | -0.221 |
| rs1468333 | 5 | 137552970 | T | C | 9.53E-14 | -0.255 |
| rs4868243 | 5 | 172643118 | G | A | 4.08E-16 | -0.361 |
| rs236349 | 6 | 36820565 | A | G | 1.01E-15 | 0.281 |
| rs9401060 (tag for rs3951016) | 6 | 118561348 | A | G | 4.04E-33 | 0.473 |
| rs1320761 | 6 | 122168138 | C | T | 1.22E-64 | 0.902 |
| rs58437978 | 7 | 35258277 | T | C | 2.61E-12 | -0.240 |
| rs180239 | 7 | 93550415 | G | C | 4.54E-21 | -0.326 |
| rs17881696 | 7 | 100493359 | G | A | 1.18E-41 | 0.578 |
| rs41748 | 7 | 116446573 | T | G | 7.14E-09 | -0.193 |
| rs11563648 | 7 | 126970046 | G | C | 4.42E-10 | -0.231 |
| rs138186803 | 7 | 130965408 | AT | A | 1.27E-16 | -0.333 |
| rs73158705 | 7 | 136576100 | A | G | 2.81E-18 | 0.393 |
| rs56233017 | 8 | 144981488 | G | A | 1.09E-15 | -0.666 |
| rs10739663 | 9 | 128278739 | A | G | 9.62E-16 | -0.266 |
| rs12576326 | 11 | 44980383 | A | G | 1.20E-12 | 0.253 |
| rs174536 | 11 | 61551927 | A | C | 1.65E-30 | 0.399 |
| rs75190942 | 11 | 128764571 | C | A | 1.19E-16 | -0.496 |
| rs2283274 | 12 | 2184466 | G | C | 7.21E-20 | -0.405 |
| rs10841486 | 12 | 20472202 | T | C | 2.98E-09 | -0.238 |
| rs4963772 | 12 | 24758480 | G | A | 3.23E-53 | -0.714 |
| rs1050288 | 12 | 27955296 | C | T | 2.74E-09 | -0.213 |
| rs1994135 | 12 | 33682405 | T | C | 7.19E-34 | 0.400 |
| rs10880689 | 12 | 37930102 | A | G | 8.10E-10 | 0.208 |
| rs867400 | 12 | 64976850 | T | C | 4.58E-19 | 0.298 |
| rs12579753 | 12 | 82219376 | C | T | 4.81E-10 | -0.246 |
| rs12889267 | 14 | 21542766 | A | G | 3.61E-20 | 0.416 |
| rs422068 | 14 | 23864804 | T | C | 1.52E-100 | 0.731 |
| rs17180489 | 14 | 72885471 | G | C | 9.15E-19 | -0.490 |
| rs1549118 | 14 | 78379684 | C | T | 4.67E-08 | 0.200 |
| rs17201923 | 14 | 85796564 | A | G | 6.55E-29 | -0.41 |
| rs4900069 | 14 | 91583373 | A | C | 5.38E-09 | 0.200 |
| rs7173389 | 15 | 73663903 | A | T | 1.31E-32 | -0.539 |
| rs3915499 | 16 | 15910743 | G | A | 1.24E-17 | 0.303 |
| rs7194801 | 16 | 65286870 | T | C | 3.58E-18 | -0.291 |
| rs79121763 | 17 | 15195279 | C | T | 7.17E-14 | -0.471 |
| rs11083258 | 18 | 25766218 | A | C | 5.51E-10 | -0.276 |
| rs61735998 | 18 | 34289285 | G | T | 2.06E-14 | -0.834 |
| rs16974196 | 19 | 40833470 | G | A | 1.11E-11 | 0.244 |
| rs12721051 | 19 | 45422160 | C | G | 5.23E-11 | -0.287 |
| rs6123471 | 20 | 36840156 | T | C | 6.63E-72 | -0.595 |
| rs17265513 | 20 | 39832628 | T | C | 1.12E-08 | 0.240 |
| rs2076028 | 22 | 39150450 | G | A | 5.45E-16 | -0.295 |

Variants that were substituted by a tag SNP are identified in parenthesis and the p-value from the original GWAS is reported. For these variants, the weight is computed as the effect of the original variant weighted by the LD in Europeans (r^2^ × β). Chromosomal positions for GRCh37. GRS, genetic risk score.
