# Supplementary material for: A genetic model of ivabradine recapitulates results from randomized clinical trials
Source: PLoS One. 2020 Jul 21;15(7):e0236193. doi: 10.1371/journal.pone.0236193 (PMC7373274; doi:10.1371/journal.pone.0236193)
Supplement: S5 Table — (DOCX) [file pone.0236193.s008.docx]

**S5 Table.** MR estimates based on 64 heart-rate associated variants and their effect on outcomes in the UK Biobank. Reported effects are per genetically predicted s.d. decrease in heart rate (1 s.d. is 11.1 bpm in the UK Biobank).

|  |  |  | **Intercept* (MR-Egger only)** | | **Causal Estimate** | |
| --- | --- | --- | --- | --- | --- | --- |
| **Exposure** | **Outcome** | **Method** | **Estimate (95% CI)** | **p-value** | **OR (95% CI)** | **p-value** |
| Heart rate reduction (1 s.d. or 11.1 bpm) | Atrial fibrillation | IVW |  |  | 1.25 (0.97, 1.62) | 0.083 |
|  |  | MR-Egger | 0.015 (-0.004, 0.034) | 0.13 | 0.85 (0.48, 1.49) | 0.57 |
|  |  | Contamination mixture |  |  | 1.54 (1.22, 1.79) | - |
|  |  | MR-PRESSO |  |  | 1.54 (1.34, 1.78) | 1.3E-07 |
|  | Heart failure | IVW |  |  | 1.02 (0.87, 1.21) | 0.77 |
|  |  | MR-Egger | 0.007 (-0.005, 0.020) | 0.60 | 0.84 (0.58, 1.21) | 0.35 |
|  |  | Contamination mixture |  |  | 1.16 (0.92, 1.43) | - |
|  |  | MR-PRESSO ^†^ |  |  | - | - |
|  | Coronary artery disease | IVW |  |  | 1.01 (0.89, 1.15) | 0.86 |
|  |  | MR-Egger | 0.004 (-0.006, 0.014) | 0.53 | 0.91 (0.68, 1.23) | 0.44 |
|  |  | Contamination mixture |  |  | 0.97 (0.87, 1.09) | - |
|  |  | MR-PRESSO |  |  | 1.01 (0.91, 1.12) | 0.86 |

* The MR-Egger estimate intercepts represent directional pleiotropy and are not converted to the OR scale because they do not have an intuitive interpretation on this scale.

^†^ The MR-PRESSO did not provide adjusted estimates as the global test did not detect significant pleiotropy (p=0.31).
