# Supplementary material for: A genetic model of ivabradine recapitulates results from randomized clinical trials
Source: PLoS One. 2020 Jul 21;15(7):e0236193. doi: 10.1371/journal.pone.0236193 (PMC7373274; doi:10.1371/journal.pone.0236193)
Supplement: S6 Table — (DOCX) [file pone.0236193.s009.docx]

**S6 Table.** MR estimates based on the effect of 64 heart-rate associated variants in external summary statistics from large GWAS consortia. Reported effects are per genetically predicted s.d. decrease in heart rate (1 s.d. is 11.1 bpm).

|  |  |  |  | **Intercept^*^ (MR-Egger only)** | | **Causal estimate** | |
| --- | --- | --- | --- | --- | --- | --- | --- |
| **Exposure** | **Dataset  (for the outcome)** | **Outcome** | **Method** | **Estimate (95% CI)** | **P value** | **OR (95% CI)** | **p-value** |
| Heart rate  reduction  (1 s.d. or  11.1 bpm) | Nielsen et al.[5] | Atrial fibrillation | IVW |  |  | 1.24 (1.00, 1.53) | 0.047 |
|  |  |  | MR-Egger | 0.167 (-0.056, 0.378) | 0.14 | 0.85 (0.49, 1.46) | 0.55 |
|  |  |  | Contamination mixture |  |  | 1.56 (1.40, 1.56) | - |
|  |  |  | MR-PRESSO |  |  | 1.36 (1.24, 1.49) | 6.3E-08 |
|  | HERMES [7] case/control | Heart failure | IVW |  |  | 1.03 (0.97, 1.12) | 0.34 |
|  |  |  | MR-Egger | -0.011 (-0.089, 0.067) | 0.79 | 1.06 (0.88, 1.29) | 0.55 |
|  |  |  | Contamination mixture |  |  | 1.12 (1.00, 1.12) | - |
|  |  |  | MR-PRESSO |  |  | - | - |
|  | CARDIoGRAMplusC4D + UKB SOFT + MiGen [3, 4] | Coronary artery disease | IVW |  |  | 1.07 (0.96, 1.19) | 0.27 |
|  |  |  | MR-Egger | 0.044 (-0.078, 0.167) | 0.45 | 0.96 (0.71, 1.29) | 0.77 |
|  |  |  | Contamination mixture |  |  | 1.00 (1.00, 1.12) | - |
|  |  |  | MR-PRESSO |  |  | 1.04 (0.95, 1.14) | 0.36 |

^*^ The MR-Egger estimate intercepts represent directional pleiotropy and are not converted to the OR scale because they do not have an intuitive interpretation on this scale.
