# Supplementary material for: A genetic model of ivabradine recapitulates results from randomized clinical trials
Source: PLoS One. 2020 Jul 21;15(7):e0236193. doi: 10.1371/journal.pone.0236193 (PMC7373274; doi:10.1371/journal.pone.0236193)
Supplement: S7 Table — (DOCX) [file pone.0236193.s010.docx]

**S7 Table.** Participant overlap between GWAS meta-analysis studies used for observational and Mendelian Randomization analyses and the UK Biobank.

| Cohort | Phenotype of interest | n cases | n controls | N UKB cases (%) | N UKB controls (%) | Ref. |
| --- | --- | --- | --- | --- | --- | --- |
| Roselli et al. | Atrial fibrillation | 65,446 | 522,744 | 16,064 (24.5%) | 334,953 (64.1%) | [6] |
| Nielsen et al. | Atrial fibrillation | 60,620 | 970,216 | 14,820 (24.4%) | 380,919 (39.3%) | [5] |
| CARDIoGRAMplusC4D | CAD | 63,746 | 130,681 | - | - | [33] |
| MIGen / CARDIoGRAM Exome | CAD | 72,868 | 120,770 | - | - | [34] |
| CARDIoGRAMplusC4D + MIGen / CARDIoGRAM Exome + UKB | CAD (UKB SOFT def.) | 83,669 * | 258,684 * | 10,801 (12.9%) | 137,914 (53.3%) | [4] |
| HERMES | Heart failure | 47,309 | 930,014 | 6,504 (13.7%) | 387,652 (41.7%) | [7] |
| Eppinga et al. | Heart rate | - | 265,046 | - | 134,251 (50.7%) | [17] |
| MEGASTROKE (European) | Stroke | 40,585 | 406,111 | - | - | [8] |
| MEGASTROKE (Trans-ethnic) | Stroke | 67,162 | 4,544,501 | - | - |  |

* We estimated these numbers based on the assumption that all participants of CARDIoGRAMplusC4D were included in the MI Genetics / CARDIoGRAM Exome study.
